# Everybody's Page

**Published:** 1890-07-12

**Authors:** 


### Everybody's Page.

Now that the season for sun-strokes is at hand it will be well for people to remember that it is quite as essential to avoid over-exertion, fatigue, or any exhaustion in hot weather as it is to keep out of the rays of a burning sun.

Those who remember all the discomforts of the port of Calais not so many years ago, when it was necessary to land in open boats, would now hardly recognise the place. The improvements which were commenced several years ago will enable Calais to become the leading centre of commerce in the north of France.

America will doubtless prove an interesting field for the archæological student when poor little Europe is dug up and exhausted of her stores. The discovery of a flint implement of the glacial period in the Tuscarawas River bears testimony, to the satisfaction of at least one expert, that man lived in the Mississippi valley before the glacial period had quitted its hold on the northern portion of the valley.

The manufacture of scents is carried on now on a large scale throughout the district of Nice. Flowers containing essence are subjected to distillation. Some of those which have no essence, such as jessamine, tuberose, and violet, are placed upon a layer of pure lard a quarter of an inch in thickness, which is spread over a tray of glass about two feet square. Several trays are piled upon one another, the flowers being changed at intervals of twelve to twenty-four hours, till the lard is sufficiently charged with perfume.

Mr. Goldwin Smith has posed recently as the champion of voluntary emigration as against State-aided emigration, so far as Canada is concerned. The idea of Government aiding emigration naturally conveys to the minds of the inhabitants of the country to which the emigrants are being sent an idea of unwarrantable interference. Such an act gives them good ground for saying, "we will not interfere with those who come of their own accord who are able to work and have a fair assurance of employment, but we object to a State invasion."

So the spleen which our forefathers looked upon as an unmitigated nuisance and of little or no use to us (and we are inclined to agree with them and throw the liver into the bargain) does some very necessary and highly useful work. The modern scientists would no doubt agree with Pliny, that people who got rid of the spleen by way of incision or burning with a hot iron would lose their laughing, but their observations have led them to believe that its retention, as an assimilator and constructor of new blood-elements, especially the white blood-globules, which act as Bow Street runners in bringing health-destroying germs to bay, is preferable to its destruction.

Anything which adds to the picturesqueness of the surroundings of our towns is welcome. Hay-making in the heart of London is a refreshing sight, and during the past week those who passed down Piccadilly must have noticed the operations in full swing in a corner of the Green Park. For the quality of the hay we cannot vouch.

We often grumble at our inability to get pure coffee and tea, and not without good cause; but perhaps we should be thankful for small mercies. In the United States samples of coffee—so-called—have shown on analysis 100 per cent. of adulteration, or an absence of the smallest particle of the berry. The inspectors, however, happily remark that adulteration is not of such frequent occurrence as it has been. This is some encouragement to future generations, but none to present victims.

Every summer we seem to hear the same melancholy stories of deaths of children from drowning, the result only too often of the carelessness of parents, who appear never to learn from experience the folly and danger of allowing children to play on the seashore out of their sight. Not the least touching part of the story of the poor little children who were playing on the rocks in Devonshire a fortnight ago is that out of the three who were saved, two fell asleep from sheer exhaustion on the sea-bound rocks during the rise and fall of the tide.

One of the latest inventions for the doing to death of that irritating creature the fly is called by the euphonious name of the "fly cemetery." The vendors assure the intending purchaser that the use of it puts an end to all interruptions of afternoon sleep and to bad language. It should therefore be of use to a great number of people. The instructions state that "when the cemetery is full it should be cremated." We were not aware of the feasibility of cremating cemeteries, but what a splendid solution of a much vexed question !

People are apt to forget that buying at very cheap shops is a mistake, which ever way we look at it. If a man advertises that he sells things much cheaper than anybody else, we may safely depend that he gets his profit somewhere. Either he gets it by selling rubbish of good appearance or else by underpaying his shop girls—a very common occurrence. Much is done by kind people who try and investigate overwork and underpay, but if the general public would lend a hand this would soon be stopped. If the public would remember that pay is often so miserable as to lead girls into dishonest and dishonourable ways of eking out their living, we are sure there would be less praise of things that are sold "dirt cheap."

